# Kinematic and dynamic gait compensations in a rat model of lumbar radiculopathy and the effects of tumor necrosis factor-alpha antagonism

**DOI:** 10.1186/ar3451

**Published:** 2011-08-26

**Authors:** Kyle D Allen, Mohammed F Shamji, Brian A Mata, Mostafa A Gabr, S Michael Sinclair, Daniel O Schmitt, William J Richardson, Lori A Setton

**Affiliations:** 1 Department of Biomedical Engineering, Duke University, 136 Hudson Hall, Box 90281, Durham, NC, USA; 2 Department of Orthopaedic Surgery, Duke University Medical Center, Orthopaedic Clinics, DUMC Box 3810, Durham, NC, USA; 3 Division of Neurosurgery, The Ottawa Hospital, 501 Smyth Road, Ottawa, ON, Canada; 4 Department of Evolutionary Anthropology, Duke University, 104 Biological Sciences Building, Box 90383, Durham, NC, USA

## Abstract

**Introduction:**

Tumor necrosis factor-α (TNFα) has received significant attention as a mediator of lumbar radiculopathy, with interest in TNF antagonism to treat radiculopathy. Prior studies have demonstrated that TNF antagonists can attenuate heightened nociception resulting from lumbar radiculopathy in the preclinical model. Less is known about the potential impact of TNF antagonism on gait compensations, despite being of clinical relevance. In this study, we expand on previous descriptions of gait compensations resulting from lumbar radiculopathy in the rat and describe the ability of local TNF antagonism to prevent the development of gait compensations, altered weight bearing, and heightened nociception.

**Methods:**

Eighteen male Sprague-Dawley rats were investigated for mechanical sensitivity, weight-bearing, and gait pre- and post-operatively. For surgery, tail nucleus pulposus (NP) tissue was collected and the right L5 dorsal root ganglion (DRG) was exposed (Day 0). In sham animals, NP tissue was discarded (*n *= 6); for experimental animals, autologous NP was placed on the DRG with or without 20 μg of soluble TNF receptor type II (sTNFRII, *n *= 6 per group). Spatiotemporal gait characteristics (open arena) and mechanical sensitivity (von Frey filaments) were assessed on post-operative Day 5; gait dynamics (force plate arena) and weight-bearing (incapacitance meter) were assessed on post-operative Day 6.

**Results:**

High-speed gait characterization revealed animals with NP alone had a 5% decrease in stance time on their affected limbs on Day 5 (*P *≤0.032). Ground reaction force analysis on Day 6 aligned with temporal changes observed on Day 5, with vertical impulse reduced in the affected limb of animals with NP alone (area under the vertical force-time curve, *P *<0.02). Concordant with gait, animals with NP alone also had some evidence of affected limb mechanical allodynia on Day 5 (*P *= 0.08) and reduced weight-bearing on the affected limb on Day 6 (*P *<0.05). Delivery of sTNFRII at the time of NP placement ameliorated signs of mechanical hypersensitivity, imbalanced weight distribution, and gait compensations (*P *<0.1).

**Conclusions:**

Our data indicate gait characterization has value for describing early limb dysfunctions in pre-clinical models of lumbar radiculopathy. Furthermore, TNF antagonism prevented the development of gait compensations subsequent to lumbar radiculopathy in our model.

## Introduction

Herniation of a lumbar intervertebral disc (IVD) can cause mechanical constriction and local inflammation of nearby neural structures, which may lead to radicular pain, numbness, weakness, and limb dysfunction [1-3]. The pathway for this pathology has been investigated in a number of pre-clinical models, including mechanical constriction of a nerve root via suture ligation, application of exogenous pro-inflammatory mediators to a nerve root, and application of autologous nucleus pulposus (NP) tissue to a nerve root [4-15]. In these models, evidence of mechanical allodynia (a hypersensitivity to non-noxious mechanical stimuli) is commonly identified, with allodynia occurring at as early as two days post-procedure and persisting out to two to six weeks [6,8-15].

Tumor necrosis factor-α (TNFα) has received significant attention as an early mediator of lumbar radiculopathy and neuropathic pain [4,6,8,13-24]. TNFα is expressed at higher levels in herniated IVD tissues relative to degeneration or cadaveric controls [17,18,25], and spinal levels of TNFα are up-regulated following proximal or distal nerve injury [26-29]. TNFα has two primary receptors, TNF receptor type I and type II; both of which have soluble and transmembrane isoforms. The functions of these receptors in TNFα signaling continues to be investigated [30], although recent evidence from TNF receptor knockout mice suggests that both TNF receptors have unique contributions to spinal cord synaptic plasticity and inflammatory pain [31]. Blocking TNF activity through either TNF sequestration or competitive inhibition of membrane-associated TNF receptors may potentially modify disease processes associated with radiculopathy [4,6,8,13,20,26-28,32-35].

Sequestration of TNFα via either an anti-TNF antibody or the soluble form of the TNF receptor is capable of modulating TNFα activity; moreover, this therapeutic strategy has demonstrated some promise in pre-clinical models of lumbar radiculopathy and peripheral neuropathy. Systemic delivery of an anti-TNF antibody (infliximab) reduced head rotations toward the affected limb, along with evidence of mechanical hypersensitivity in a rat model [6,8,32]. Both soluble TNF receptor type I and etanercept (a fusion protein of soluble TNF receptor type II and the Fc component of the human immunoglobulin G1) have been shown to attenuate thermal and mechanical hypersitivities in rat radiculopathy models [13,20,28,34,35]. For the human condition, however, the efficacy of TNF antagonism is more controversial. A single intravenous infusion of infliximab did not improve patients with disc herniation relative to placebo control at three months or one year in the FIRST II clinical study [36,37]. However, more recently, epidural delivery of etanercept spaced at two-week intervals was reported to improve patient pain scores relative to saline placebo at three months follow-up in a small patient cohort [38]. Thus, there is continued interest in local administration of TNF antagonists for lumbar radiculopathy. In this study, we investigate the ability of a TNF antagonist, the soluble form of TNF receptor type II (sTNFRII), to reverse gait compensations and hypersensitivities in a rat model lumbar radiculopathy.

Behavioral changes observed in pre-clinical models of lumbar radicular pain may relate to painful symptoms observed in human subjects. Patients with low back pain and sciatica report fear of movement and substantial decreases in activity levels [39], and recently, patients with lumbar spinal stenosis reported significantly lower activity levels than both control subjects and patients with either knee or hip osteoarthritis [40]. Patients with lumbar radiculopathy have also been found to use reduced walking velocities, shorter stride lengths, and increased periods of double limb support [41]. The impact of lumbar radiculopathy on locomotion is relatively unknown in pre-clinical models, despite being of clinical relevance. Moreover, changes in nociception (allodynia and hyperalgesia) may not necessarily be related to changes in rodent gait [9,42]. Instead, gait compensations may relate to spontaneous pain generation or limb dysfunction following nerve injury. In prior work, mechanical hypersensitivity and gait compensations were found to follow unique time scales in a rat surgical model of lumbar radiculopathy [9]. While affected limb hypersensitivity was elevated throughout the four-week experiment, imbalanced and asymmetric gait patterns were observed within the first post-operative week and began to normalize on Week 2 [9]. These quantitative assessments of rodent gait characteristics may provide important information on the potential of a pharmaceutical to correct limb compensations following lumbar radiculopathy, and to date, no studies have investigated the ability of TNF antagonism to block the development of limb dysfunction and gait compensations following lumbar radiculopathy in the rat.

In this study, we expand upon the description of gait compensations following lumbar radiculopathy in the rat through the use of quantitative measures of gait kinematics, dynamics, and weight distribution. Moreover, we investigate the ability of a TNF antagonist, sTNFRII, to reverse gait abnormalities and hypersensitivities observed within the first post-operative week. Our results demonstrate that rats with lumbar radiculopathy use imbalanced, asymmetric gaits which serve to decrease the vertical impulse experienced by the affected limb. Furthermore, the application of a TNF antagonist ameliorated evidence of hypersensitivity, imbalanced weight distribution, and gait abnormalities, further suggesting that TNF plays a key role in the initiation of gait compensations following lumbar radiculopathy in the rat.

## Materials and methods

### Experimental design

Eighteen Sprague-Dawley rats (three months, male) were acquired from Charles Rivers Laboratory. Animals were acclimated in the housing facilities at Duke University for one week prior to pre-operative behavioral evaluations (t = -4 to -3 days, denoting three to four days prior to the surgical procedure). On Day 0, animals received one of the surgical procedures described below. Following surgery, rats were monitored to ensure the animal was weight-bearing on the operated limb. On Day 5, animals were evaluated for spatiotemporal gait characteristics and mechanical sensitivity. On Day 6, animals were evaluated for dynamic gait characteristics and weight bearing. Animals were sacrificed on Day 7. All procedures described herein were approved by the Duke University Institutional Animal Care and Use Committee (IACUC).

### Surgical model

Lumbar radiculopathy was examined using a surgical model described previously [9]. Briefly, rats were anesthetized with intraperitoneal pentobarbital (60 mg/kg) and maintained on 2% isoflurane via mask inhalation for the duration of the procedure. Tail nucleus pulposus (NP) tissue was collected from a caudal intervertebral disc, and the right L5 dorsal root ganglion (DRG) was exposed via a partial unilateral laminotomy and medial facetectomy. At this point, animals were allotted to one of three groups as follows: 1) tail NP tissue was discarded (Sham, *n *= 6); 2) autologous tail NP tissue was placed on the exposed right L5 DRG (NP alone, *n *= 6); or, 3) autologous tail NP tissue was placed on the exposed right L5 DRG, along with 20 μg of rh-sTNFRII (Abcam, Cambridge, MA, USA, 18.9 kDa) in 25 μL PBS delivered locally at the exposed L5 DRG (NP and sTNFRII, *n *= 6). The exposed DRG was closed using 3-0 vicryl sutures for fascia and 3-0 nylon sutures for skin closure. The tail surgical site was closed via a single layer of 3-0 nylon sutures. Since all surgical groups received a partial medial facetectomy and unilateral laminectomy, subcutaneous injection of buprenorphine HCl (Buprenex, 0.02 mg/kg, Reckitt Benckinse Healthcare, Hull, England) was provided intra-operatively and every 12 hours out to Day 2 (four total doses). Days 5 and 6 were selected as the post-operative behavioral assessment time points to provide a reasonable recovery period for post-operative pain, while remaining within a time period where gait differences have been previously described between sham and NP placement surgeries [9]. The concentration of sTNFRII was selected based upon reports for an ability of sTNFRII to attenuate inflammatory events in intervertebral disc cells, wherein IC50 values were reported to fall between 20 to 35 nM for antagonizing TNFα-induced nitric oxide and prostaglandin E2 release [26].

### Geometric and temporal gait descriptors

To assess geometric and temporal descriptors of rodent gait, rats were placed in a custom-built gait arena (5'6" × 1'6") preoperatively and again on Day 5. The arena is composed of a glass floor, three transparent acrylic sides, a black acrylic back, black acrylic top, and a mirror oriented at 45° underneath the arena floor. This setup allows for simultaneous viewing of foot-placements in the sagittal and ventral planes. When a rat passes through the middle four feet of the arena, a single high-speed video camera is manually triggered to capture the rat's movement (Phantom V4.2, 200 frames per second; Vision Research, Wayne, NJ, USA). Rats were allowed to freely explore the arena until five acceptable videos were acquired (<20 minutes. per animal); all trials contained a minimum of two complete gait cycles and a consistent velocity (less than 15% velocity change about the mean). Videos of a grid pattern attached to the arena's floor were also acquired, allowing for the conversion of video pixels to geometric coordinates during post-processing.

Using a custom MATLAB code, gait videos were analyzed for velocity. Briefly, each video frame (grayscale) was subtracted from an image without a rat in the arena and then thresholded to obtain a binomial image. The centroid of the animal was obtained for frames containing the entire torso (regionprops, MATLAB); velocity and direction of travel were then calculated from these positional data. The position and video frame of foot-strike and toe-off events were determined through by-hand digitization using the DLTdataviewer [9,43,44]. The first frame describing ground contact and the last frame describing ground contact for the hind limbs could be visualized in the sagittal plane. For each event, the geometric position of the foot in the ventral plane was marked using the digitization software. Pixel coordinates and frame numbers were converted into geometric and time variables. The following data were calculated for each trial: stride length, step width, percentage stance time, and gait symmetry. Percentage stance time (also known as limb duty factor) is defined as the amount of time a limb is in stance for a given stride, or mathematically as stance time divided by stride time [45]. Gait symmetry is defined as the offset between left and right foot-strikes in a limb pair for a given stride, or mathematically as the time between left and right foot-strike events divided by the time between two left foot-strike events [45].

Velocity differences between treatment groups (preoperative, sham, NP alone, NP + sTNFRII) were assessed using a one-way ANOVA with a *post-hoc *Newman-Keuls test. Since step width, stride length, and percentage stance time can show weak to strong correlations to an animal's selected velocity, a generalized linear modeling (GLM) approach was used to account for a linear dependence on trial velocity, followed by a *post-hoc *Newman-Keuls test. For temporal descriptors, rats typically ambulate with balanced, symmetric gaits. This gait pattern is represented mathematically by a difference between the left and right percentage stance times of 0 and a gait symmetry variable of approximately 0.5. A shift in either of these variables would indicate a shift away from a balanced, symmetric gait. For the statistical analyses of percentage stance time imbalance and gait asymmetry, each group is compared to the mathematical definitions for balanced, symmetric gait using a repeated measures t-test with a Bonferroni correction; differences among treatment groups were analyzed using a one-way ANOVA with a *post-hoc *Newman-Keuls test.

### Ground reaction force analysis

To assess ground reaction forces, rats were placed in a custom-built force plate arena (4'6" × 6") preoperatively and again on Day 6. This arena is composed of three acrylic sides, a black acrylic side (back of the arena), and a medium density fiberboard floor. At its center, a 1" × 6" section of the floor is isolated and attached to an overload protected portable Hall-effect-based force plate (6" × 6" × 1.16", ±2.45 N x- and y-axis, +4.9 N z-axiz, 200 Hz collection speed; Advanced Mechanical Technology, Inc., Watertown, MA, USA), calibrated as previously described [46]. The direction of forces during locomotion were defined such that +Fx indicates propulsive forces in the direction of travel (-Fx indicates braking forces), +Fy indicates mediolateral forces directed toward the animal's midline for both the right and left hind limb, and +Fz indicates vertical force perpendicular to the contact area.

Multiple trials of the left and right hind limb ground reaction forces were acquired for each rat during a 25-minute period. When a rat strikes the isolated section of the floor, one of two video cameras was manually triggered to capture the rat's movement (Phantom V4.2; Sony Handycam HDR-XR200V, Sony USA, New York, NY, USA). During post-processing, these videos were used to verify that the foot was in complete contact with the isolated section of floor only; these videos were not used to quantify gait metrics as described for geometric and temporal gait descriptors. Videos where only a portion of the foot landed on the isolated section of floor were excluded from the analysis, since ground reaction forces were not entirely directed at the force plate. For this reason, trial numbers were unbalanced among groups: 24 left and 31 right foot trials for pre-operative, 19 left and 21 right foot trials for sham controls, 14 left and 17 right foot trials for NP alone, and 20 left and 18 right foot trials for NP and sTNFRII. Force plate data for these trials were imported into MATLAB and passed through a 25 Hz low-pass filter to reduce noise.

Force curves were normalized to the animal's body weight. Normalized curves were then generalized into the following measures for the statistical analysis [47]: 1) Fx ground reaction forces were described by peak braking force (Max F_-x_), peak propulsive force (Max F_x_), braking phase impulse (I_-x_), propulsive phase impulse (I_x_), percentage braking time (t_-x_); 2) Fy ground reaction forces were described by the first peak force (Max F_y,0-50%_), second peak force (Max F_y,50-100%_), and mediolateral impulse (I_y_); and, 3) Fz ground reaction forces were generalized by the peak vertical force (Max F_z_) and vertical impulse (I_z_). A two-factor ANOVA followed by a *post-hoc *Newman-Keuls test was used to compare differences among treatment groups and between the affected and contralateral limb. All reported statistics were conducted on weight-normalized data sets as described above; however, in order to present meaningful units for comparison against other studies, non-normalized data are presented in the results section and in data tables.

### Weight distribution

Hind limb weight distribution was determined preoperatively and again on Day 6 using an incapacitance meter (IITC, Inc., Woodland Hills, CA, USA). Briefly, an incapacitance meter consists of two scales and specialized caging to encourage a rearing posture in the research animal. Weight on the left and right limb was acquired during five-second intervals (five trials per rat). These data were converted into weight distribution by dividing the weight on the right limb by the total weight for both hind limbs. Weight distribution imbalance was determined using a repeated measures t-test with a *post-hoc *Bonferroni correction (imbalance ≠ 50%); differences among treatment groups were analyzed using a one-way ANOVA and a *post-hoc *Newman-Keuls test.

### Mechanical sensitivity

Mechanical paw withdrawal thresholds were determined preoperatively and again on Day 5 using an up-down protocol described by Chaplan and coworkers [48]. Briefly, rats were placed in a wire-bottom cage and allowed to acclimate to the caging for 30 minutes. Von Frey filaments (Stoelting, Wood Dale, IL, USA) were then applied to the plantar surface of rat's hind paws. If paw withdrawal was observed, the next smallest filament was applied; if paw withdrawal was not observed, the next largest filament was applied. Using this up-down protocol, the 50% paw withdrawal threshold can be approximated; this threshold represents the mechanical force where paw withdrawal and stimulus tolerance are equally likely. A two-factor ANOVA followed by a *post-hoc *Newman-Keuls test was used to compare differences among treatment groups and between the affected and contralateral limb.

## Results and discussion

### Temporal gait characteristics

Velocities tended to increase post-operatively, with animals receiving NP and sTNFRII walking at faster velocities than pre-operative controls (*P *<0.001) and animals receiving NP alone (*P *= 0.018). Pre-operative speeds were 29.8 ± 1.0 cm/sec; at Day 5 post-operation, sham animals walked at 34.9 ± 2.1 cm/sec, NP alone animals walked at 32.6 ± 1.5 cm/sec, and animals with NP and sTNFRII walked at 39.0 ± 1.8 cm/sec (mean ± standard error). Percentage stance times are known to decrease with speed; at these respective speeds, affected limb percentage stance time for each group were 72.4 ± 0.6 (pre-operative), 70.2 ± 1.0 (sham), 67.3 ± 0.9 (NP alone), and 65.7 ± 1.0 (NP and sTNFRII), and contralateral limb percentage stance times were 72.6 ± 0.7 (pre-operative), 69.6 ± 1.2 (sham), 71.4 ± 1.0 (NP alone), and 66.0 ±1.1 (NP and sTNFRII).

**Figure 1:**
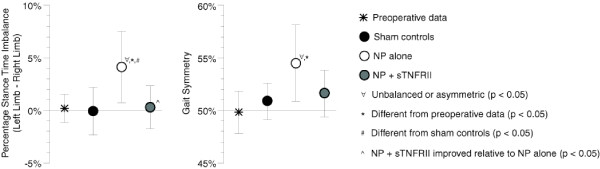
**Unbalanced, asymmetric gait in rats with lumbar radiculopathy (five days post-operation)**. Unbalanced, asymmetric gaits were observed in animals with NP alone (∀, *P *<0.032), while all other groups did not differ significantly from the mathematical definition of balanced, symmetric gait. The percentage stance time imbalance observed in animals with NP alone differed from both preoperative (*, *P *= 0.025) and sham controls (#, *P *= 0.013), and this imbalance was improved in animals receiving NP and sTNFRII relative to animals with NP alone (^, *P *= 0.012). Gait symmetry of animals with NP alone was also significantly different from pre-operative controls (*, *P *= 0.009). Data are presented as mean ± standard error.

Animals with NP alone used imbalanced, asymmetric gaits (*P *≤ 0.032, Figure 1), while animals in all other groups did not differ significantly from balanced, symmetric gait. The percentage stance time imbalance observed in animals with NP alone was significantly different from the gait pattern of both preoperative (*P *= 0.025) and sham controls (*P *= 0.013); this percentage stance time imbalance was also significantly improved in animals receiving NP and sTNFRII treatment relative to animals with NP alone (*P *= 0.012). Gait symmetry of animals with NP alone was also significantly different from pre-operative controls (*P *= 0.009) and tended to be different from sham controls (*P *= 0.055); similar to percentage stance time imbalance, gait symmetry tended to improve in animals with NP and sTNFRII relative to animals with NP alone (*P *= 0.062). The imbalanced gait pattern of animals with NP alone favors the affected limb by significantly reducing affected limb stance time relative to the contralateral limb, while the asymmetric pattern increases the time from contralateral-to-affected limb foot-strike and reduces the time from affected-to-contralateral limb foot-strike.

**Figure 2:**
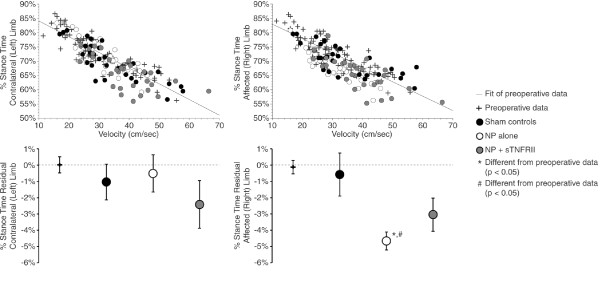
**Affected and contralateral percentage stance time in rats with lumbar radiculopathy (five days post-operation)**. A significant correlation between percentage stance time and velocity is observed in walking rats (upper left and right); raw data with a linear fit of the pre-operative data are shown. As velocity increases, percentage stance time decreases in both the affected (right) and contralateral (left) limb. After accounting for this correlation, decreased affected limb percentage stance times (bottom right), but not contralateral limb stance times (bottom left) can be identified in animals with NP alone relative to pre-operative and sham controls (*P *= 0.010, *P *= 0.013, respectively). While sTNFRII treatment improved stance time imbalance resulting from NP application to the L5 DRG (see Figure 1), improvement in the stance time balance in the sTNFRII treated rats appears to result from a relative decrease in both the affected and contralateral limb stance times relative to pre-operative and sham controls.

Imbalanced stance times in the NP alone group were primarily driven by a decrease in affected limb stance time at a given velocity, not by an increase in the contralateral limb stance time (Figure 2). In the affected limb, animals with NP alone had reduced percentage stance time at a given velocity relative to preoperative and sham controls (*P *= 0.010, *P *= 0.013, respectively); no differences between groups were observed in the contralateral limb stance time. While sTNFRII treatment improved stance time imbalance resulting from NP application to the L5 DRG, improvement in the stance time balance in the sTNFRII treated rats appears to result from a relative decrease in both the affected and contralateral limb stance times relative to pre-operative and sham controls (non-significant). It is not immediately clear whether the tendency to reduce percentage stance time in both affected and contralateral limbs of animals with NP and sTNFRII is indicative of injury, as percentage stance time changes may also result from rodent growth, changes in muscle strength, or changes to the percentage stance time-velocity relationship. However, the relative difference between sham controls and animals with NP and sTNFRII may indicate that some injury persists following NP application to the L5 DRG that cannot be altered by TNF antagonism.

In this study and in a previous study [9], imbalanced, asymmetric gaits for rats with lumbar radiculopathy are reported within the first post-operative week. These measures reflect the synchronization of two limbs in a limb pair, and as such, both symmetry and percentage stance time imbalance can reflect syncopations that are indicative of limping-like behaviors in both the quadrupedal gait of rodents and the bipedal gait of humans. These temporal shifts in the sequence of gait events occur very rapidly in rodents and are undetectable with the human eye. For example, the stance times for a given limb of a three-month old rat are approximately 0.2 to 0.6 seconds during walking; thus, our reported 5% shift in percentage stance time would represent a 0.01 to 0.03 second change in the raw stance time. Thus, reports that gross visual inspection of rodent gait show the affected limb to be weight bearing during ambulation are reasonable and understandable [49-53]; however, detailed quantification of rodent gait through high-speed image analysis does reveal a repeatable pattern of imbalanced, asymmetric gait at one-week post-operative. In this study, further verification that high-speed methods at 200 Hz/fps can accurately detect gait abnormalities in a rat model of lumbar radiculopathy is provided; moreover, these same abnormalities can altered in our model through TNF antagonism.

In patients with lumbar spinal stenosis, an increase in double limb support time is observed relative to a control population [41]. For a bilateral injury, an increase in double limb support would reduce single limb support phases for both limbs, while the gait pattern observed in the hind limbs of our rat model of unilateral lumbar radiculopathy would reduce single limb support phases in the affected limb only. While the shifts in raw percentage stance time vary, both gait patterns serve to reduce single limb support in the affected limbs, though gaits with increased double limb support may be balanced and symmetric. Thus, it is possible that the imbalanced, asymmetric gait compensations observed during the early phase of lumbar radiculopathy may distinguish a focal unilateral pathology of lumbar radiculopathy from the generalized pain syndrome of lumbar spinal stenosis and IVD degeneration. Further work is needed to verify this hypothesis.

The temporal analysis presented herein focuses on changes within the hind limb pair only. In quadruped gait, use of the forelimbs may also be adapted to compensate for hind limb injury. In prior work, changes in the fore-limb pair were found to be less substantial than changes in the hind limb pair [44], and thus, our data and methods are focused on identifying hind limb compensations. Moreover, changes in stance time imbalance within the hind limb pair were found to be primarily driven by changes in the affected limb, and not necessarily through a change in contralateral limb stance time (Figure 2). While gait abnormalities in the fore limbs may be of interest for describing compensations resulting from lumbar radiculopathy in the rat, hind limb compensations are likely to be of greater magnitude and more easily detected.

### Geometric gait characteristics

**Figure 3:**
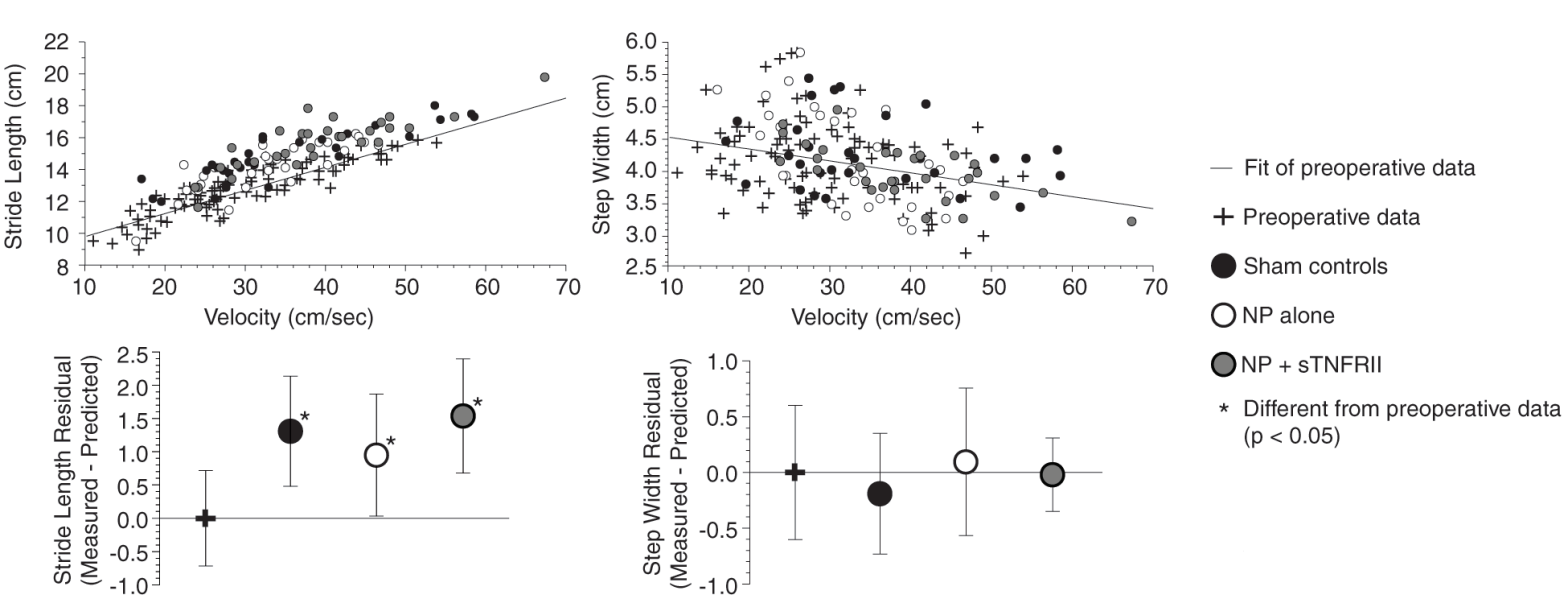
**Stride length and step widths in rats with lumbar radiculopathy (five days post-operation)**. A significant correlation between stride length and velocity is observed in walking rats (upper left); raw data with a linear fit of the pre-operative data are shown. As velocity increases, stride length increases. Even after accounting for this correlation, stride lengths were found to increase in sham controls, animals with NP alone, and animals with NP and sTNFRII (*, lower left, *P *<0.016). A weaker, but important, correlation is also seen between step width and velocity (upper right); again, raw data with a linear fit of the pre-operative are shown. Here, as velocity increases, step widths narrow; however, after accounting for this correlation, no differences were observed in step width between groups (lower right). Data in lower graphs are presented as mean ± standard error.

As predicted, stride lengths increased and step widths narrowed with an increase in velocity (Figure 3). While stride lengths at a given velocity were longer post-operatively in all groups (*P *≤0.016), no differences between post-operative groups were observed for stride lengths or step width. Changes in stride length between pre-operative and post-operative time points may be due to rodent growth or changes in muscle strength between the two time-points.

Stride lengths have been previously reported in a rat model of lumbar radiculopathy using a foot-printing method [54]. In this prior work, stride lengths were compared for the left and right limb, and statistical differences were not found. In our approach, stride length differences are investigated after accounting for a stride length dependence on animal velocity. Over a velocity range of 10 to 70 cm/sec, stride lengths vary by 80% based upon velocity alone. By using a GLM, the effects of a velocity covariate were incorporated into the statistical mode; however, even with this methodology, stride length changes that associate with lumbar radiculopathy were not identified in this study or in prior work [9].

Changes in geometric gait variables, including stride length and step width, are less likely than temporal variables to describe gait compensations due to limb injury in the rodent [9,44,55,56]. Moreover, the analysis of geometric data in rodents is complicated by changes in rodent size and strength and a dependence upon velocity -- a variable that is uncontrollable in rodents without the use of a treadmill and gait training. Humans with either back or leg pain tend to take shorter strides; however, this type of compensation has been difficult to identify in rodent models of musculoskeletal injury [44,55,56]. It is not evident as to why this inconsistency occurs: It may be due to differences between quadrupedal and bipedal gait, conditioned through evolution as a manner of masking injury, lost within variability caused by velocity changes and animal growth, altered by habituation to the gait test, or affected by stress associated with limb injury in the rat. The reasons for this inconsistency are not clear and are far beyond the scope of this study. However, the data, herein and in past reports, clearly highlight the challenge of using geometric data to measure gait compensations associated with musculoskeletal injury in rodent models. Temporal data may be more valuable in describing gait compensations in the rodent due to musculoskeletal injury and provide a more direct translation between that of the quadruped animal model and the human condition.

### Weight distribution and ground reaction forces

**Figure 4:**
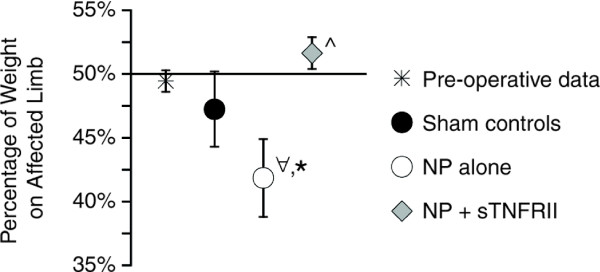
**Weight-bearing characteristics of rats with lumbar radiculopathy (six days post-operation)**. Rats with NP alone had unbalanced weight-distribution, supporting significantly less weight on their affected limb relative to their contralateral limb (∀, *P *= 0.048). This weight-distribution was significantly different from pre-operative controls (*, *P *= 0.022). Rats with NP and sTNFRII treatment had improved weight distribution relative to rats with NP alone (^, *P *= 0.005). Data are presented as mean ± standard error.

Weight distribution imbalance was observed in animals receiving NP alone (*P *= 0.048, Figure 4). These animals supported significantly less weight on the affected limb, differing significantly from pre-operative controls (*P *= 0.022). Weight distribution imbalance was not observed in the pre-operative, sham, or NP and sTNFRII groups. Moreover, animals with NP and sTNFRII had improved weight distribution relative to animals with NP alone (*P *= 0.005).

**Figure 5:**
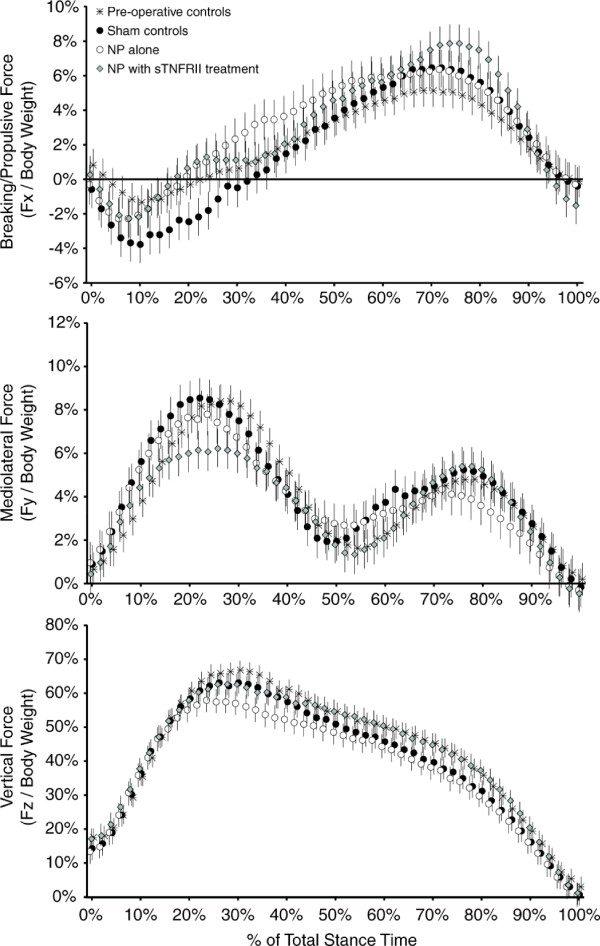
**Normalized force curves for the affected limbs of rats with lumbar radiculopathy (six days post-operation)**. Ground reaction forces represented in these plots were created by normalizing forces to body weight and time to the total limb stance time. Fx curves show a breaking phase (Force <0), during which, the limb is loaded and resists the translation of mass forward. Fx curves also show a propulsion phase (Force >0) during which the limb is generating push-off forces in the direction of travel. Fy force curves show a peak between approximately 0 to 50% of stance time, during which the limb is being loaded as mass is transferred onto the limb for single limb support. A second peak is seen from approximately 50 to 100% of stance time; here, the limb is generating push-off forces that translate mass toward the contralateral limb. Fz force curves show a loading phase from 0 to 30% of stance time, a support phase from 30 to 80% of stance time, and an unloading phase from 80 to 100% of stance time. In general, the shape of the force curves were as expected for each group; however, differences between generalized terms for each group were observed (See Table 2). Each data point is presented as mean ± 95% confidence interval.

Representative ground reaction curves for the affected limbs are shown in Figure 5. To account for differences in the total stance time among groups and between trials, data presented in Figure 5 were binned and averaged across animals within each treatment group [57]. Generalized ground reaction force data are presented in Table 1. Body weights increased in the sham, NP alone, and NP and sTNFRII groups relative to pre-operative data (*P *<0.001), and animals with NP alone tended to weigh less than sham controls at one week post-operation (*P *= 0.055).

**Table 1:** Generalized gait dynamics descriptors for a rat model of lumbar radiculopathy

Body weight	Pre-operative	Sham	NP Alone	NP + sTNFRII
Mass (g)	285 ± 4	348 ± 6*	325 ± 9*	340 ± 11*
**Affected limb**	**Pre-operative**	**Sham**	**NP Alone**	**NP + sTNFRII**
Max F_z _(mN)	2,156 ± 65	2,069 ± 79	1,877 ± 88	2,016 ± 85
I_z _(mN-s)	416.6 ± 25.5	463.2 ± 31.0	325.4 ± 34.5^∀,#^	417.9 ± 33.5
Max F_-x _(mN)	-56 ± 15	-170 ± 18*	-111 ± 20	-142 ± 20*
I_-x _(mN-s)	-3.5 ± 1.6	-15.3 ± 2.0*	-6.7 ± 2.2	-10.3 ± 2.1
t_-x _(% stance time)	20.7 ± 2.8	30.4 ± 3.4	21.2 ± 3.8	27.7 ± 3.7
Max F_x _(mN)	177 ± 18	204 ± 22	240 ± 25*	256 ± 24*
I_x _(mN-s)	24.0 ± 3.2	28.6 ± 3.9	34.0 ± 4.3	32.9 ± 4.2
Max F_y,0-100% _(mN)	280 ± 19	301 ± 23	301 ± 25	286 ± 25
Max F_y,50-100% _(mN)	182 ± 12	203 ± 15	185 ± 16	176 ± 16
I_y _(mN-sec)	38.9 ± 2.2	46.2 ± 4.3	38.0 ± 4.2	40.4 ± 2.8
**Contralateral limb**	**Pre-operative**	**Sham**	**NP Alone**	**NP + sTNFRII**
Max F_z _(mN)	2,154 ± 74	2,079 ± 83	1,984 ± 97	1,914 ± 81
I_z _(mN-s)	439.5 ± 29.0	475.6 ± 32.6	488.3 ± 38.0	440.3 ± 31.8
Max F_-x _(mN)	-79 ± 17	-160 ± 19*	-124 ± 22	-116 ± 19
I_-x _(mN-s)	-5.7 ± 1.9	-13.3 ± 2.1*	-11.6 ± 2.4	-6.7 ± 2.0
t_-x _(% stance time)	23.4 ± 3.2	30.4 ± 3.6	22.7 ± 4.2	18.1 ± 3.5
Max F_x _(mN)	169 ± 21	263 ± 23*	274 ± 27*	304 ± 23*
I_x _(mN-s)	23.0 ± 3.6	33.2 ± 4.1	40.2 ± 4.7*	42.4 ± 3.9*
Max F_y,0-100% _(mN)	285 ± 21	304 ± 24	276 ± 28	231 ± 23
Max F_y,50-100% _(mN)	167 ± 14	210 ± 16	182 ± 18	184 ± 15
I_y _(mN-sec)	40.5 ± 2.6	45.2 ± 4.3	49.1 ± 5.7	36.2 ± 3.6

∀, *P *<0.05 vs. contralateral control limb

*, *P *<0.05 vs. pre-operative control

#, *P *<0.05 vs. sham control

Data presented as mean ± standard error

Animals with NP alone had a lower vertical impulse (I_z_) in their affected limbs relative to their contralateral limb (*P *= 0.009). In addition, affected limb I_z _was lower in animals with NP alone relative to sham controls (*P *= 0.029) and tended to be lower than pre-operative controls (*P *= 0.069); treatment with sTNFRII also tended to improve affected limb I_z _relative to NP alone (*P *= 0.061). While differences between treatment groups were not observed for peak vertical force (Max F_z_), Max F_z _did follow a similar profile toward reduced values in rats with NP alone, (non-significance, ANOVA *P*-value = 0.411). In our rats, peak vertical force occurred at the end of limb loading, near the time of contralateral limb toe-off (approximately 25 to 35% of the affected limb stance time). Until contralateral limb toe-off, which represents the transition from double limb support to single limb support on the affected limb, the F_z _curves for each treatment group are very similar. It is after the peak vertical force where the force curves appear to diverge. Thus, our data indicate that vertical force changes due to lumbar radiculopathy in the rat are occurring primarily when the affected limb is in single limb support and possibly during affected limb unloading, but not necessarily during limb loading.

Maximum braking force (Max F_-x_) in the affected limb of sham animals and animals with NP and sTNFRII was higher than preoperative controls (*P *= 0.002, *P *= 0.020, respectively), and Max F_-x _in the contralateral limb of sham animals was higher than preoperative controls (*P *= 0.004). Braking impulse (I_-x_) in both the affected and contralateral limb of sham animals was higher than preoperative controls (*P *= 0.005, *P *= 0.028, respectively). Differences between treatment groups were not found for braking time (t_-x_). Maximum propulsive force (Max F_x_) in the affected limb of animals with NP alone or NP and sTNFRII was higher than preoperative controls (*P *= 0.039, *P *= 0.007, respectively), and Max F_x _in the contralateral limb of sham animals, animals with NP alone, and animals with NP and sTNFRII were higher than preoperative controls (*P *= 0.014, *P *= 0.005, *P *<0.001, respectively). Propulsive impulse (I_x_) in the contralateral limb of animals with NP alone and animals with NP and sTNFRII were higher than preoperative controls (*P *= 0.022, *P *= 0.004, respectively).

While differences in braking/propulsion curves were identified, changes that associate specifically with lumbar radiculopathy in the rat are challenging to decipher. The braking and propulsion changes observed were between post-operative measures and pre-operative controls, without a clear separation between post-operative groups. It is plausible that these changes are occurring in conjunction with the increased stride lengths and/or changes in rodent size, limb length, and strength. Regardless, braking and propulsion changes that associate with lumbar radiculopathy in the rat could not be clearly identified within our animals.

No differences between groups was observed for the mediolateral force curves (Max F_y,0-50%_, Max F_y,50-100%_, I_y_).

While force plate analysis has been used to investigate motor deficits following nerve resection and spinal cord injury in rats [58,59], we believe this is the first study to investigate gait dynamics associating with lumbar radiculopathy in a rat model. Among measures of gait dynamics, vertical impulse appears to be strongly affected by lumbar radiculopathy in the rat. While vertical force as a percentage of body weight is much higher in bipeds, the change in the vertical force curve due to lumbar radiculopathy in the rat is relatively consistent with injury compensations found in bipedal gait; and like percentage stance time imbalance and gait symmetry, vertical impulse can reflect differences between the affected and contralateral limb in both quadrupeds and bipeds. In addition, these metrics follow a similar profile: Rats in the NP alone group at one-week tend to have changes in stance time imbalance, gait symmetry, and vertical impulse relative to pre-operative, sham, and contralateral controls; and, the application of sTNFRII largely ameliorated the effects of NP placement alone. Hence, gait metrics that are capable of describing differences between the affected and contralateral limb in time may be the preferred measures of gait compensations associating with lumbar radiculopathy in the rat.

### Mechanical sensitivity

**Figure 6:**
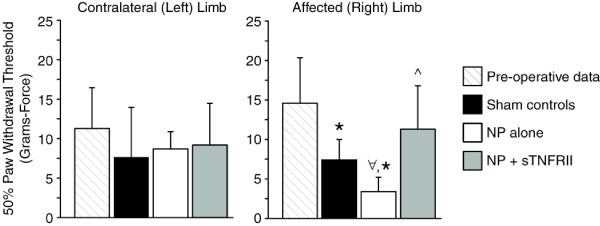
**Mechanical sensitivity in the affected and contralateral limb of rats with lumbar radiculopathy (five days post-operation)**. Rats with NP alone had significant decreases in the mechanical withdrawal threshold of their affected limb relative to their contralateral limb (∀, *P *= 0.032) and pre-operative controls (*, *P *= 0.001). Sham controls also had significant decreases in their mechanical withdrawal threshold relative to pre-operative controls (* *P *= 0.030). Rats receiving NP and sTNFRII treatment had improved mechanical withdrawal thresholds relative to rats with NP alone (^, *P *= 0.013). Data presented as mean + standard error.

Affected limb mechanical withdrawal thresholds decreased in the sham and NP alone groups relative to pre-operative values (*P *= 0.03, *P *= 0.001, respectively, Figure 6). While animals with NP alone had the lowest mean withdrawal threshold, this threshold did not significantly differ from animals with sham surgery (*P *= 0.129) or from contralateral controls (*P *= 0.08). Animals receiving NP and sTNFRII had improved paw withdrawal thresholds relative to NP alone (*P *= 0.013). Differences among surgical groups were not observed for the contralateral limb mechanical paw withdrawal threshold.

The use of anti-TNF therapeutics as a treatment for lumbar radiculopathy is controversial. While no single mediator can be consistently linked to painful radiculopathy, TNF has been a primary focus for both clinical and pre-clinical studies [4-8,10-13,15,51,53,60]. In pre-clinical animal models, there are multiple examples of TNF antagonism attenuating aspects of lumbar radiculopathy, including the reduction of thermal and mechanical hypersensitivities, and reduction of nerve root edema and inflammation [4,6,8,10,13,15]. However, a single systemic administration of TNF antagonists in a clinical trial failed to attenuate leg and back pain associated with IVD herniation at three months or one year [36,37]. The reasons for the discrepancy between pre-clinical models and clinical results are not yet clear. It is possible that a single systemic administration of a TNF antagonist may not achieve an effective dose at the injury site; in a small cohort of patients, epidural delivery of etanercept spaced at two-week intervals was reported to improve patient pain scores relative to saline placebo at three months follow-up [38]. In addition, it is possible that TNFα is highly involved in the early stages of lumbar radiculopathy, but may not have as central a role in the chronic condition [8]. As a result, well-controlled pre-clinical studies that specifically target the acute phase of IVD herniation may demonstrate efficacy for TNF antagonism, while clinical studies that include the large variation of IVD herniations and disease stages seen in the patient population may fail to demonstrate an analogous efficacy.

## Conclusions

Prior studies of rat models of lumbar radiculopathy have reported either normal gait characteristics based upon visual inspection [49-53] or mild gait compensations based upon foot-printing methods [54]. Here, we report the first high-speed force plate analysis of rat gait following lumbar radiculopathy and couple this with a high-speed video characterization of rat gait. Dynamic gait compensations align well to spatiotemporal data, with data indicating that the affected limb is protected through reduction of the affected limb's relative stance time and vertical impulse. Moreover, delivery of sTNFRII at the right L5 DRG simultaneous with NP placement on the same DRG reduced hypersensitivity in the affected limb, improved rodent weight distribution and returned gait metrics to near-preoperative levels. At less than one week post-operative, the reported gait compensations in animals with NP alone coincide with mechanical hypersensitivity in the affected limb and imbalanced weight distribution, demonstrating that gait kinematics and dynamics can be useful for measuring dysfunction following lumbar radiculopathy in the rat. While gait analyses can be labor intensive, the focused use of a few metrics of gait and weight bearing have the potential to describe symptomatic behaviors indicative of lumbar radiculopathy in rat. The behavioral metrics with the potential to associate with lumbar radiculopathy in the rat, based upon our data, are summarized in Table 2. In conclusion, our data demonstrate that gait characterization can be used to describe limb dysfunction occurring during the early stages of lumbar radiculopathy in the rat, and that, gait metrics and dynamics may be valuable measures in pre-clinical studies evaluating drug effectiveness.

**Table 2:** Behavioral metrics with potential to associate with lumbar radiculopathy in the rat

	Affected limb vs. Contralateral limb (NP alone group)	NP alone vs. Pre-operative data (one week post-op)	NP alone vs. Sham controls (one week post-op)	NP alone vs. NP + sTNFRII (one week post-op)
% Stance time imbalance	Unbalanced	3.9%	4.7%	3.8%
				
(High-speed videography, 200 fps)	(*P *= 0.032)	(*P *= 0.025)	(*P *= 0.013)	(*P *= 0.012)
Gait symmetry	Asymmetric	5.3%	3.6%	2.9%
(High-speed videography, 200 fps)	(*P *= 0.031)	(*P *= 0.008)	(*P *= 0.055)	(*P *= 0.062)
Affected limb vertical impulse	162.9 mN-sec	91.2 mN-sec	137.8 mN-sec	137.8 mN-sec
(Force plate, 200 Hz)	(*P *= 0.009)	(*P *= 0.069)	(*P *= 0.029)	(*P *= 0.029)
Weight bearing	Unbalanced	7.6%	5.4%	5.4%
(Incapacitance meter)	(*P *= 0.048)	(*P *= 0.022)	(*P *= 0.052)	(*P *= 0.052)
Affected limb mechanical sensitivity	5.3 gF	11.2 gF	4.0 gF	4.0 gF
(von Frey test)	(*P *= 0.148)	(*P *= 0.001)	(*P *= 0.129)	(*P *= 0.129)

From our analysis, the measures of animal behavior in this table tended to yield the highest significance level at one week after lumbar radiculopathy in the rat. Data are presented as the mean difference between groups (top) with the associated *P*-value (below). In general, these metrics tend to describe differences between the affected and contralateral sides of the animal (column 1), show changes relative to preoperative and sham controls (column 2 to 3), and tend to improve with the application of sTNFRII at the time of surgery (column 4).

## Abbreviations

ANOVA: analysis of variance; BSA: bovine serum albumin; DRG: dorsal root ganglion; GFAP: glial fibrillary acidic protein; Iba1: ionized calcium binding adaptor molecule 1; IVD: intervertebral disc; I_-x_: braking impulse; I_x_: propulsive impulse; I_y_: mediolateral impulse; I_z_: vertical impulse; Max F_-x_: peak braking force; Max F_x_: peak propulsive force; Max F_y,0-50%_: first peak mediolateral force; Max F_y,50-100%_: second peak mediolateral force; Max F_z_: peak vertical force; NP: nucleus pulposus; PBS: phosphate buffered saline; sTNFRII: soluble tumor necrosis factor receptor type II; TNF: tumor necrosis factor; t_-x_: braking time (as a percentage of stance time).

## Competing interests

The authors declare that they have no competing interests.

## Authors' contributions

KDA conducted all gait and behavioral analyses, organized the experimental design, and drafted the manuscript with the assistance of LAS. MFS, BAM and MAG performed the animal surgeries and local drug delivery. SMS was responsible for sTNFRII drug preparation. DOS assisted in the analysis and interpretation of gait data and also provided technical assistance for the collection of ground reaction forces. WJR assisted in the development of the surgical model. WJR and LAS assisted in the conception and design of the experiment and the analysis and interpretation of the data. All authors have read and approved the final manuscript.
